# Long-Term Outcome of Autologous Hematopoietic Stem Cell Transplantation (AHSCT) for Acute Myeloid Leukemia (AML)- Single Center Retrospective Analysis

**DOI:** 10.1007/s12253-017-0266-7

**Published:** 2017-06-28

**Authors:** Grzegorz Helbig, Anna Koclęga, Krzysztof Woźniczka, Małgorzata Kopera, Sławomira Kyrcz-Krzemień

**Affiliations:** School of Medicine in Katowice, Department of Hematology and Bone Marrow Transplantation, Medical University of Silesia, Dąbrowski street 25, 40-032 Katowice, Poland

**Keywords:** Acute myeloid leukemia, Autologous hematopoietic stem cell transplantation, Relapse, Outcome

## Abstract

For patients with acute myeloid leukemia (AML) in complete remission without an acceptable HLA donor, the autologous hematopoietic stem cell transplantation (AHSCT) may remain a therapeutic option as remission consolidation, however its role is still a subject of continued debate. One hundred and twenty patients who underwent AHSCT for AML were included in this retrospective single center analysis. The procedure was performed over a 19 years period and transplanted patients were in first complete remission (CR1; *n* = 109) or in second CR (CR2; *n* = 11). The median age at transplant was 37 years (range 18–64). The source of stem cells was bone marrow (*n* = 61; 50.8%), peripheral blood (*n* = 36; 30%) and bone marrow with peripheral blood (*n* = 23; 19.2%). The median time from AML diagnosis to AHSCT was 0.8 year (range 0.3–4.4) and the median follow-up after AHSCT for surviving patients was 12.8 years (range 3.1–20.5). The median LFS was 1.1 year. The probability of LFS calculated at 5 years and 10 years after transplantation was 28% (95%CI, 22%–32%) and 21% (95%CI, 18%–24%), respectively. The last relapse occurred 14.8 years after AHSCT and among patients who survived >2 years, 28.4% (27/95) had leukemia recurrence. The median OS was 1.7 years. The probability of OS after 5 years and 10 years was 29% and 22%, respectively. There was a tendency for increased LFS for patients younger than 50 years at transplant if compared to older population. AHSCT for AML was safe with acceptable toxicity profile. Leukemia recurrence remained the leading cause of death.

## Introduction

An intensive combined chemotherapy including anthracycline and cytarabine remains a mainstay of induction treatment for medically fit and younger (<60 years) patients with acute myeloid leukemia (AML) producing a complete remission (CR) rate ranges from 60% to 70% [1]. The continuation of post-remission therapy seems to be mandatory in order to prevent relapse which may occur in virtually all patients after CR achievement [2]. The optimal post-remission treatment is still a matter of debate and may include repeated cycles of high-dose cytarabine, autologous hematopoietic stem cell transplantation (AHSCT) and allogeneic hematopoietic stem cell transplantation (AlloHSCT). The choice of post-remission therapeutic strategy should be guided by several factors associated with disease (e.g. cytogenetics), patient (e.g. performance status) or transplant (e.g. donor availability). The strongest anti-leukemic effect is associated with AlloHSCT, however it is counterbalanced by high non-relapse treatment-related mortality [3].

The role of AHSCT as a post-remission therapy for AML patients remains unclear. The toxicity of AHSCT seems to be comparable with that of intensive chemotherapy. The main concern is still associated with a high leukemia recurrence rate which usually occurs within the first 2 years after transplant with good prognosis for those who survived after this period [4]. Nevertheless, the cumulative relapse incidence at 10 years was 16% in the recent report of the European Society for Blood and Marrow Transplantation (EBMT). Older patient age, peripheral blood as a source of stem cells and M0, M6 and M7 leukemia subtypes were found to be associated with higher relapse rate in the multivariate analysis [5].

Overall, the patients autografted in CR1 had superior leukemia-free survival (LFS) and lower leukemia recurrence, but not overall survival (OS) if compared with those treated with intensive chemotherapy. The summary of numerous studies has demonstrated that AHSCT should be offered for younger patients in the favorable and intermediate cytogenetic risk groups, however its optimal role requires further investigations [6].

Herein we report the outcome of adults patients with AML who underwent AHSCT in our institution over a 19-year period.

## Material and Methods

### Patients and Procedures

One hundred and twenty patients who underwent AHSCT for AML were included in this retrospective single center analysis. The procedure was performed between years 1990 and 2009 and transplanted patients were in first complete remission (CR1) or in second CR (CR2). The median age at transplant was 37 years (range 18–64) and 13.3% of patients were >50 years at the time of procedure. There was a slight male predominance (51.6%). 56% of AML patients were diagnosed before the year 2000. The diagnosis of AML was established according to the French-American-British (FAB) classification. The distribution between studied patients was as follows: M0 in 1.6% (*n* = 2), M1 in 19.1% (*n* = 23), M2 in 34.1% (*n* = 41), M4 in 34.1% (*n* = 41), M5 in 8.3% (*n* = 10), M6 in 1.6% (*n* = 2) and M7 in 0.8% (*n* = 1). The patients with M3 were excluded from analysis. The cytogenetic/molecular results were available for merely 27 patients (22.5%) and 8/27 (29.6%) subjects were assigned to favorable [6 pts. with t(8;21) and 2 pts. with inv.(16)] and 14/27 (51.8%) to intermediate (all with normal diploid karyotype without FLT3-ITD mutation) and 5/27 (18.5%) to adverse risk category (3 pts. with detectable FLT3-ITD mutation and 2 pts. with monosomy 7). Prior myelodysplastic syndrome was diagnosed in 9.1% of patients. The vast majority of patients were transplanted in CR1 (90.8%; *n* = 109) whereas the remaining 11 subjects (9.2%) had CR2. The source of stem cells was bone marrow (*n* = 61; 50.8%), peripheral blood (*n* = 36; 30%) and bone marrow with peripheral blood (*n* = 23; 19.2%). Graft ex vivo purging was not performed. The assessment of minimal residual disease (MRD) in collected product using flow cytometry or molecular tests was not a routine practice at that time in our center. Most transplants were performed before the year 2000 (52.5%; *n* = 63) and the commonest conditioning regimen consisted of busulfan and cyclophosphamide (84.1%; *n* = 101). Details were shown in Table 1.

**Table 1:** Study patients characteristics

Patients characteristics	Number of patients: 120
Age at transplant (y; median; range)	37 (18–64)
>60 y	3 (2.5%)
Between 50 and 59 y	13 (10.8%)
<50 y	104 (86.6%)
Gender	
female/male	58/62
Date of AML diagnosis >2000 y	53 (44.1%)
Prior MDS	11 (9.1%)
FAB classification	
M0	2 (1.6%)
M1	23 (19.1%)
M2	41 (34.1%)
M4	41 (34.1%)
M5	10 (8.3%)
M6	2 (1.6%)
M7	1 (0.8%)
AML status at transplant	
CR1	109 (90.8%)
CR2	11 (9.2%)
Year of transplant >2000 y	57 (47.5%)
Source of stem cells	
Bone marrow	61 (50.8%)
Peripheral blood	36 (30.0%)
Bone marrow and peripheral blood	23 (19.2%)
Type of conditioning	
Chemotherapy-based	120 (100%)
Busulfan/cyclophosphamide	101 (84.1%)
Busulfan and cytarabine	4 (3.3%)
Etoposide/cytarabine/cyclophosphamide	15 (12.5%)
Number of transplanted CD34-positive cells (median; range)	2.02 (0.39–13.3)
Time to ANC recovery >0.5 G/L (days; range)	21 (11–120)
Time to PLT recovery >50 G/L (days; range)	45 (11–390)
Number of RBCs transfusions (units; median; range)	3 (0–33)
Number of PLT transfusions (units; median, range)	5 (0–42)
Death to day +100 after AHSCT	5 (4.1%)

*AML* acute myeloid leukemia, *ANC* absolute neutrophil count, *AHSCT* autologous hematopoietic stem cell transplantation; *BM* bone marrow, *CR* complete remission, *FAB* French-American-British, *MDS* myelodysplastic syndrome, *PB* peripheral blood; *PLT* platelets, *RBC* red blood cells

### Statistical Methods

The probability OS and LFS were calculated according to Kaplan-Meier estimate. The Kruskal-Wallis test was used to compare more than two independent groups of variables. The probability of LFS was defined as the time from transplant to relapse or death in CR. All calculations were made from the date of transplantation. Comparisons between the variables were carried out by log-rank test. Statistical significance was defined at a *P* value <0.05. Transplant-related mortality (TRM) was defined as death within 100 days after AHSCT not related to disease or relapse. Data analysis was censored at the time of AlloHSCT in 7 patients who relapsed.

## Results

The median time from AML diagnosis to AHSCT was 0.8 year (range 0.3–4.4) and the median follow-up after AHSCT for surviving patients was 12.8 years (range 3.1–20.5). Twenty three patients were alive at the time of analysis. The median number of transplanted CD34-positive cells was 2.02 (range 0.39–13.3). Absolute neutrophil count (ANC) >0.5 G/L and platelet (PLT) count >50 G/L were achieved after median of 21 days (range 11–120) and 45 days (11–390), respectively. The fastest ANC recovery was observed in patients transplanted from PB (med. 17 days; range 11–37), if compared with PB/BM (med. 23 days; range 11–42) and BM (med. 29 days; range 11–120) *p* < 0.001. In regard to PLT count, the fastest recovery was demonstrated for PB (med. 18 days; range 11–160), if compared with BM (med. 55 days; range 11–390) and PB/BM (med. 79 days, range 20–180) *p* = 0.003.

Five patients died within the first 100 days after transplantation; all deaths were due to severe infectious complications. More than half of transplanted patients suffered from moderate stomatitis and pharyngitis (*n* = 66). 23 patients had fever of unknown origin. Bacteremia was present in 15 subjects and 33% of them developed severe bilateral pneumonia leading to death. The other complications included herpes zoster reactivation (*n* = 7), proctitis (*n* = 6), hepatitis (*n* = 6) and urinary tract infections (*n* = 3).

The median LFS was 1.1 year. The probability of LFS calculated at 5 years and 10 years after transplantation was 28% (95% confidence interval [95%CI], 22%–32%) and 21% (95%CI, 18%–24%), respectively. The last relapse occurred 14.8 years after AHSCT and among patients who survived >2 years, 28.4% (27/95) had leukemia recurrence.

**Fig. 1:**
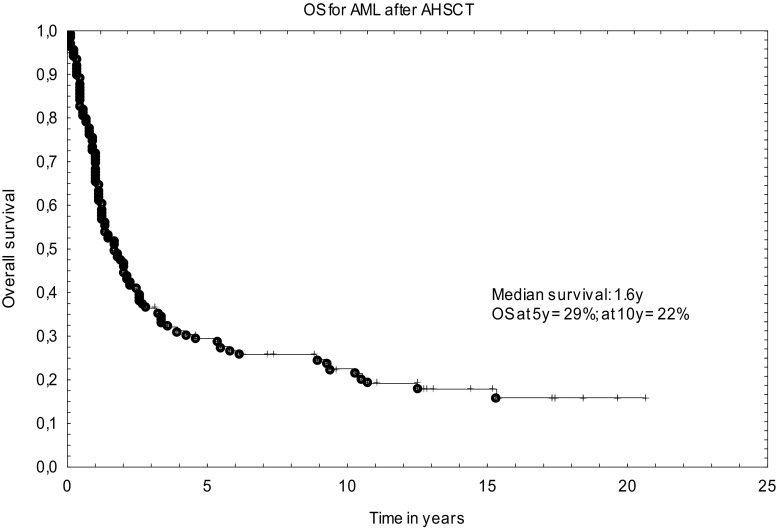
Overall survival curve for AML patients after autologous transplantation

**Fig. 2:**
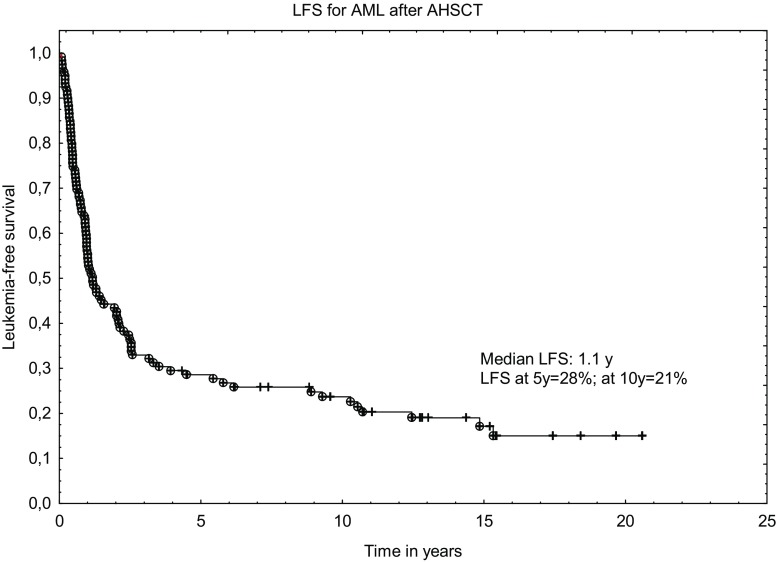
Leukemia-free survival curve for AML patients after autologous transplantation

The median OS was 1.7 years. The probability of OS after 5 years and 10 years was 29% (95%CI, 25%–33%) and 22% (95%CI, 19%–25%), respectively (Figs. 1 and 2). No factor was found to have a significant impact on LFS and OS in univariate analysis (Table 2). There was a tendency for better LFS in patients younger than 50 years at transplant if compared with older population (32% [95%CI 28–36] vs 6% [95%CI 0–12]; *p* = 0.06).

**Table 2:** Prognostic factors in univariate analysis

Factor	5y OS (95%CI)	5y LFS (95%CI)
Age		
≥ 50y	12 (4–20)	6 (0–12)
< 50y	32 (28–36)	32 (28–36)
	*p* = 0.19	*p* = 0.06
Gender		
Female	27 (22–32)	28 (22–34)
Male	31 (26–36)	28 (23–33)
	*p* = 0.9	*p* = 0.88
Date of AML diagnosis		
< 2000y	30 (25–35)	28 (23–32)
≥ 2000y	28 (22–34)	25 (19–31)
	*p* = 0.89	*p* = 0.93
Prior MDS		
Yes	22 (9–35)	22 (9–35)
No	30 (26–34)	29 (25–33)
	*p* = 0.62	*p* = 0.68
FAB classification		
M1	30 (20–40)	30 (20–40)
M2	36 (29–43)	36 (29–43)
M4	24 (17–31)	23 (17–29)
M5	20 (8–32)	12 (1–21)
	*p* = 0.36	*p* = 0.74
AML status at transplant		
CR1	30 (26–34)	29 (25–33)
CR2	11 (1–21)	11 (1–21)
	*p* = 0.23	*p* = 0.33
Year of transplant		
≥ 2000y	26 (20–32)	26 (20–32)
< 2000y	31 (26–36)	30 (25–35)
	*p* = 0.93	*p* = 0.77
Source of stem cells		
BM	33 (27–39)	31 (25–37)
PB	24 (17–31)	22 (15–29)
BM + PB	27 (18–36)	25 (16–34)
	*P* = 0.92	*p* = 0.24

*AML* acute myeloid leukemia, *BM* bone marrow, *CR* complete remission, *FAB* French-American-British, *MDS* myelodysplastic syndrome, *PB* peripheral blood

Cytogenetic and molecular results were available only for 27 patients and probably for that reason, no difference between risk categories in terms of LFS rates was demonstrated. The probability of LFS calculated at 5 years after AHSCT for favorable, intermediate and adverse risk groups was following: 37%, 30% and 20%, respectively (*p* = 0.9).

Seven patients who relapsed after AHSCT were proceeded to AlloHSCT from matched unrelated donors. Three of them are alive in CR whereas the remaining 4 died due to post-transplant complications (Table 3).

**Table 3:** Patient undergoing AlloHSCT after relapse post AHSCT

Patient ID	Gender	AML FAB	Age at AHSCT (y)	Time between AHSCT and AlloHSCT (y)	Time from ALLOHSCT (y)	Current status
KB	F	2	22	14	1.7	Alive in CR
TC	M	5	31	0.3	11.4	Alive in CR
KK	F	4	19	0.7	NA	Dead
LM	M	4	37	2.5	NA	Dead
RS	M	1	30	0.5	NA	Dead
RW	F	1	43	1.6	5.2	Alive in CR
EP	F	2	48	0.4	NA	Dead

*AML* acute myeloid leukemia, *AHSCT* autologous hematopoietic stem cell transplantation, *AlloHSCT* allogeneic hematopoietic stem cell transplantation, *CR* complete remission, *FAB* French-American-British, *F* female, *M* male, *NA* not applicable

## Discussion

For patients with AML without an acceptable HLA donor, the AHSCT may remain an option for post-remission consolidation. In the present study, we analyzed the results of our single institution experience in AHSCT for AML patients. A total of 120 patients with a median age of 37 years were autografted over a 19 year period. The vast majority of patients were transplanted in CR1 (91%) and 23 survivors were followed for an average of 13 years with a maximum follow-up of more than 20 years. The median LFS and OS survivals were 13 months and 19 months, respectively and they were comparable with those reported by others [7]. It should be stressed that the last relapse occurred almost 15 years after AHSCT and 27 (28%) patients had late (>2 years) leukemia recurrence.

Historically, the use of bone marrow (BM) as a source of stem cells was associated with difficulties in stem cell collection and delayed hematopoietic recovery [8]. The introduction of peripheral blood (PB) progenitor cells as a source of stem cells allowed for better collection yield, faster engraftment and reduction of infection risk [9]. 70% of patients (*n* = 84) from our study received BM-derived stem cells, however almost 30% of them (*n* = 23) yielded an insufficient number of cells and required an extra growth factor -stimulated PB collection. In fact, the fastest ANC and PLT recovery was demonstrated for patients transplanted from PB if compared with those receiving stem cells form BM and BM/PB (*p* < 0.001 and *p* < 0.003, respectively). The infection rate did not differ between groups (data not shown). The LFS and OS curves were also comparable.

If we compare our results with those reported by Collison et al. [7], we found a comparable probability of LFS and OS at 5 years. Moreover, these authors found age and karyotype as predictors of poor outcome. Adverse cytogenetic risk also correlated with a decreased probability of LFS and OS in a large EBMT study published recently [5]. In contrary, no cytogenetic abnormality nor any other tested factors were identified as the predictors for LFS and OS in our study. However, it should be mentioned that majority of our patients had unavailable cytogenetic data. The reason for that can partially be related to the fact that about 60% of patients were diagnosed before the year 2000 when the tests were not routinely performed in our country.

It has been demonstrated that patients who were leukemia-free more than 2 years after transplant less likely had disease recurrence [4, 7]. However, this statement is becoming false in the light of the recently published paper by Czerw et al. [5]. The recurrence incidence was 16% at 10 years and this finding was in line with that presented by other groups [10]. To conclude, disease relapse seems to be the leading cause of death in autografted AML patients. The same is also true for our study cohort. All fatal outcomes were due to leukemia recurrence with subsequent resistance to chemotherapy. The long-term survival was seen only in patients who underwent AlloHSCT, but it also was not a rule. It was reported by others that higher probability of relapse was associated with PB as a source of stem cells, probably due to graft contamination with leukemic cells [5]. No difference between PB and BM in terms of disease relapse was found in our study.

Despite several published studies a definitive place for AHSCT in AML patients in CR1 is a subject of continued debate. A large prospective randomized trial of remission consolidation with AHSCT versus chemotherapy for AML has been published recently. 517 patients were included over a 11-year period. This study has demonstrated reduced relapse rate (58% vs 70%) and tendency to better LFS (38% vs 29%) in AHSCT arm. OS were comparable (44% vs 41%) between arms at 5 years from randomization. 17% of patients in AHSCT arm if compared with 25% in chemotherapy arm required a rescue procedure with AlloHSCT [11]. A continued sub-analysis of AML patients aged 40–60 years showed comparable OS following AHSCT and AlloHSCT in the intermediate cytogenetic risk group, however LFS was significantly increased in the latter [12]. Treatment-related mortality after AHSCT was 4% [11]. The above-mentioned results compare well with those presented by Keating et al. The authors compared LFS and OS rates for patients receiving AlloHSCT vs AHSCT for AML in CR1 and they did not find any difference between studied groups. A cumulative incidence of relapse at 5 years was significantly higher for AHSCT than AlloHSCT whereas a significantly lower treatment-related mortality rate at 5 years was demonstrated for the former group [13]. The German AML96 Study has found a significantly better OS in autografted patients in the intermediate cytogenetic risk group if compared with chemotherapy and AlloHSCT arms (62% vs 41% vs 44%, respectively) [14]. Interestingly, the results of cytogenetic studies did not influence the post-transplant outcome in the Keating study [13] and the reason for that is difficult to explain.

We present long-term data of AML patients who were autografted in CR1/CR2. All those patients did not have a matched related- or unrelated donor at the time of decision. One should keep in mind that more than 50% of patients were diagnosed and transplanted before the year 2000 when the availability of volunteer donors was limited, especially in Eastern and Central Europe. The major concern of our study was associated with missing cytogenetic data. As a consequence, unselected AML population was proceeded to transplant and decision was not directed by genetic risk. Surely, it had a great impact on data interpretation and transplant outcome. Moreover, MRD assessment in collected product was not a routine practice and the infusion of MRD-positive cells was likely. A proportion of our patients was in CR2 at transplant. Interestingly, the LFS and OS were comparable between subjects transplanted in CR1 and CR2. Of note is, that the number of patients in CR2 was small (<10%) to draw conclusions. A vast majority of our patients were less than 60 years at transplant. The results of AHSCT for older AML population are scarce. It was demonstrated that such patients had the 3-year LFS and OS of 28% and 39%, respectively [15].

One should be aware that the current policy of our center is to perform AHSCT only in AML patients being assigned to favorable cytogenetic risk group with MRD negativity of transplanted product. The patients with intermediate and adverse cytogenetic/molecular features are proceeded to AlloHSCT. Of note is, that in our center there are no patients left without transplantation after completion of post-remission consolidation. The probability of OS at 2 year after AlloHSCT for AML patients assigned to intermediate/adverse risk groups in our center is about 60% (data not shown).

In summary, the autografting for AML was safe with acceptable post-transplant toxicity. If compared with chemotherapy, AHSCT reduced relapse risk and gives better LFS with comparable OS. The major concern of AHSCT in AML patients results from high rate of leukemia recurrences. It seems reasonable to elaborate the strategies which might prevent disease relapse. The introduction of minimal residual disease assessment may allow for early introduction of preventive strategies [3]. AlloHSCT should be offered for relapsed patients who achieved subsequent CR.
